# Quality of Novel Coronavirus Related Health Information over the Internet: An Evaluation Study

**DOI:** 10.1155/2020/1562028

**Published:** 2020-08-06

**Authors:** Ashish Joshi, Fnu Kajal, Soumitra S. Bhuyan, Priya Sharma, Ashruti Bhatt, Kanishk Kumar, Mahima Kaur, Arushi Arora

**Affiliations:** ^1^City University of New York Graduate School of Public Health and Health Policy, New York, NY, USA; ^2^Urban Local Bodies, Government of India, New Delhi, India; ^3^Edward J. Bloustein School of Planning and Public Policy, Rutgers University, New Brunswick, NJ, USA; ^4^Foundation of Healthcare Technologies Society, New Delhi, India; ^5^Icahn School of Medicine at Mount Sinai, New York, NY, USA

## Abstract

**Background:**

The novel coronavirus disease (COVID-19) has spread globally from its epicenter in Hubei, China, and was declared a pandemic by the World Health Organization (WHO) on March 11, 2020. The most popular search engine worldwide is Google, and since March 2020, COVID-19 has been a global trending search term. Misinformation related to COVID-19 from these searches is a problem, and hence, it is of high importance to assess the quality of health information over the internet related to COVID-19. The objective of our study is to examine the quality of COVID-19 related health information over the internet using the DISCERN tool.

**Methods:**

The keywords included in assessment of COVID-19 related information using Google's search engine were “Coronavirus,” “Coronavirus causes,” “Coronavirus diagnosis,” “Coronavirus prevention,” and “Coronavirus management”. The first 20 websites from each search term were gathered to generate a list of 100 URLs. Duplicate sites were excluded from this search, allowing analysis of unique sites only. Additional exclusion criteria included scientific journals, nonoperational links, nonfunctional websites (where the page was not loading, was not found, or was inactive), and websites in languages other than English. This resulted in a unique list of 48 websites. Four independent raters evaluated the websites using a 16-item DISCERN tool to assess the quality of novel coronavirus related information available on the internet. The interrater reliability agreement was calculated using the intracluster correlation coefficient.

**Results:**

Results showed variation in how the raters assigned scores to different website categories. The .com websites received the lowest scores. Results showed that .edu and .org website category sites were excellent in communicating coronavirus related health information; however, they received lower scores for treatment effect and treatment choices.

**Conclusion:**

This study highlights the gaps in the quality of information that is available on the websites related to COVID-19 and study emphasizes the need for verified websites that provide evidence-based health information related to the novel coronavirus pandemic.

## 1. Introduction

Novel coronavirus (COVID-19) belongs to the large family of coronavirus like SARS-CoV [1]. On December 31, 2019, China reported a cluster of pneumonia cases in people associated with the seafood wholesale market in Wuhan, Hubei Province. On January 7, 2020, Chinese health authorities confirmed that this cluster was associated with a novel coronavirus, 2019-nCoV. The World Health Organization (WHO) initially named this coronavirus as the 2019-novel coronavirus (2019-nCoV) on January 12, 2020. The WHO officially named the disease as coronavirus disease 2019 (COVID-19), and the Coronavirus Study Group (CSG) of the International Committee proposed to name the new coronavirus as SARS-CoV-2 on February 11, 2020 [2]. Although cases were originally reported to be associated with exposure to the seafood market in Wuhan, current epidemiologic data indicate that person-to-person transmission of 2019-nCoV is occurring [3].

The WHO declared the COVID-19 virus outbreak a pandemic on March 11, 2020. Most people infected with SARS-CoV-2 experience mild to moderate respiratory illness and might recover without requiring special treatment. As of May 31, 2020, there have been nearly 5 million total coronavirus cases and 362,786 deaths [4]. There is an urgent need to understand the epidemiology and evolution of this outbreak, as well as control strategies to stop the transmission [5].

The COVID-19 outbreak has posed critical challenges for public health, research, and medical communities [6]. Every outbreak provides an opportunity to gain valuable information, some of which are associated with a limited window of opportunity. More and more individuals are relying on the internet for latest information on the pandemic, which makes it necessary to ensure credibility and accuracy of these sources [7]. The quality of internet-based health information is extremely variable as there are no mandatory standards for peer review of websites with the potential to provide wrong information to individuals seeking self-care [8]. Assessing credibility of health information over the internet is essential [9].

A previous study suggested three basic requirements for quality information: (1) the information presented in a manner free from propaganda or disinformation (objectivity); (2) the information is a complete, not partial, picture of the subject (completeness); and (3) all aspects of the information are given, and the information is not restricted to present a particular viewpoint (pluralism) [10].

Public health experts worry that the spread of COVID-19 worsened by misinformation. Experts worry dissemination of false or even unsafe information at an alarming rate. The World Health Organization (WHO) described “massive infodemic,” citing an overabundance of reported information, accurate and false, about the virus that makes it hard for people to find trustworthy sources and reliable guidance when they need it [11]. Experts suggest that false or even dangerous information about what can protect individuals during the pandemic is being disseminated at an alarming rate. Facebook, Twitter, and Google said they were working with the WHO to address “misinformation.” A lack of credible, easy-to-access information for individuals has led to some pursuing unorthodox approaches to health care that can be harmful. Stores are selling hand sanitizers that do not meet the Centers for Disease Control and Prevention (CDC) guidelines and may not be effective, and bad actors are hawking homeopathic products that can harm unwitting individuals [12].

Several tools exist to evaluate the quality of health information on the internet. Three tools that are widely used include the HON (Health on Net) Code, the JAMA benchmarks, and the DISCERN tool [13]. The DISCERN tool is the only tool currently available online for which substantive validation data are publicly available. The first section of this tool evaluates the reliability of the information, and the second section considers the quality of the information on treatment choices. Five-point Likert scales ranging from one (definite no) to five (definite yes) accompany these items. The final question assesses the overall rating of the publication on a five-point Likert scale ranging from one (low quality with serious or extensive shortcomings) to five (high quality with minimal shortcomings) [14]. Low scores indicate poor quality of information, and high scores indicate good quality. The DISCERN tool has an internal consistency of Cronbach's *α* (0.777) [15].

The DISCERN questionnaire is a reliable instrument and can be used as an assessment tool to evaluate health information not only by health professionals but also by patients and the general population [16]. The DISCERN tool is relatively easy to use as demonstrated in previous studies which also reported an excellent interrater reliability.

The objective of our study was to evaluate the quality of COVID-19 related health information over the internet using the DISCERN tool.

## 2. Methods

### 2.1. Selection of Websites

**Figure 1:**
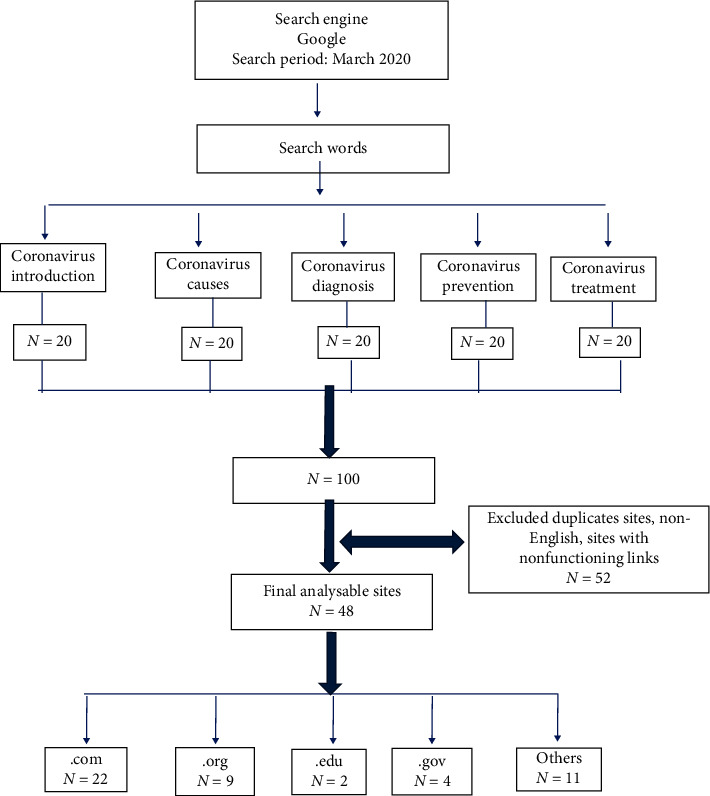
Description for the internet search for novel coronavirus related health information.

In March of 2020, we used Google's search engine to assess the quality of coronavirus related information over the internet (Figure 1). The keywords used in this search included “Coronavirus,” “Coronavirus causes,” “Coronavirus diagnosis,” “Coronavirus prevention,” and “Coronavirus management.” We recorded the first 20 websites that appeared in the results of various keyword searches on Google, generating 100 URLs. Prior studies have shown the importance of the first 20 websites as a source of the most reliable content [17]. Duplicate websites excluded from this search, allowing analysis of unique websites only. Additional exclusion criteria included scientific journals, nonoperational links, nonfunctional websites (where the page was not loading, was not found, or was inactive), and websites in languages other than English.

### 2.2. Criteria Development for Rating Websites

Previous studies [15, 18] have identified nine criteria for website assessment, including the following:

1. Source (such as credentials, conflicts of interest, and biases)
2. Disclosure (statement of purpose and profiling)
3. Accuracy
4. The correctness of material
5. Statement of source
6. Levels of evidence
7. Disclaimers
8. Link content (evaluated according to selection, architecture, content, and back linkages)
9. Peer-review mechanisms (content reviewed by experts and colleagues in the related area)

Quality of health information on the internet must determine accuracy of the material, relevancy, topic clarity, and level of evidence in the form of citations of peer-reviewed material [17, 19]. In our study, we used the DISCERN tool (http://www.discern.org.uk), which was designed for use by consumers of online health information and does not require previous knowledge of the subject [20]. It is a validated rating tool used by health consumers or professionals alike [21]. The tool is a 16-item questionnaire used to assess the quality of health information on a website and to help healthcare providers and consumers to evaluate any website containing health information [22].

We divided the 16 items of the DISCERN tool in six categories: Relevance, Objectives, Information Credibility, Treatment Choices, Treatment Effect, and Prevention and Management (Table 1). Each category scored on a Likert scale of 1 to 5. A higher score indicated that the website contains more useful and appropriate information while a lower score indicated lack of information in the identified categories. The content on these forty-eight websites identified in our search was considered accurate and complete only if the website provided the following information on novel coronavirus: risk factors for virus transmission, diagnosis, treatment effect, and treatment choices along with information on disease prevention and management.

### 2.3. Evaluation of Websites

In accordance with the URL suffixes, the websites were categorized as .edu, .com, .org, .gov, and others (for all remaining domains). Four raters belonging to diverse educational backgrounds also frequent internet users independently assigned a score between 1 and 5 to each of forty-eight websites in the six DISCERN categories (Relevance, Objectives, Information Credibility, Treatment Choices, Treatment Effect, and Prevention and Management). For final assessment, an average of the combined scores used. A high total score denotes that the website is providing high-quality consumer health information.

### 2.4. Statistical Analysis

Descriptive statistics in the form of means and standard deviations for continuous variables and percentage distributions for categorical variable (web domain categories and DISCERN tool groups) are reported. Interrater reliability (IRR) analysis was performed to assess the level of agreement among raters in scoring websites within each domain category (.com, .edu, .org, .gov, and others) and by the DISCERN tool group (Relevance, Objectives, Information Credibility, Treatment Choices, Treatment Effect, and Prevention and Management) (Tables 2 and 3). We have also reported the *p* value for comparison between average DISCERN score and web domain groups using analysis of variance (ANOVA) (Table 2). We calculated the average DISCERN group score of the four raters and assessed whether the quality of information differed between domain groups and each DISCERN group score using ANOVA (Table 4). Comparison of scores was done across different categories of the DISCERN tool, including objectives, relevance, information credibility, treatment choices, treatment effect, prevention, and overall quality of the publication. Additional analysis was performed to compare the DISCERN scores across the five domain categories (.com, .edu, .gov, .org, and others). All tests were performed using SAS9.4.

## 3. Results

The initial search resulted in 100 websites, of which 52 excluded because they either contained insufficient information or were not in English, had nonfunctional links, or were duplicates. The remaining 48 unique websites were included in the final evaluation. Forty-six percent (*n* = 22) of the websites had .com website extension, 19% (*n* = 9) had .org website extension, and 23% (*n* = 11) had other website extension categories (Figure 1).

**Figure 2:**
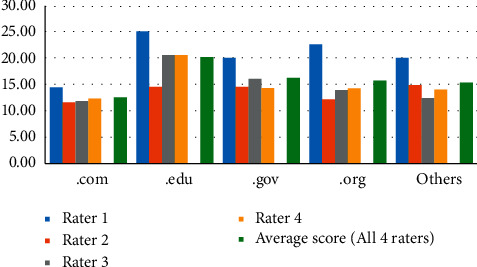
Average DISCERN score by website domain categories.

Results showed that of all the raters, highest discern scores by one of the raters were given to website extension .edu (25) and .org (22.56). Low DISCERN scores were seen for both .edu and .com website categories (Figure 2).

Table 2 represents the average DISCERN score in each web domain by each of the four raters and the overall average rating of the four raters. Of the four raters, three raters consistently gave highest DISCERN scores to the website extension category .edu. An intracluster correlation coefficient value between 0– 30 demonstrates poor interrater reliability. Overall, the scoring reliability between raters remained low for each web domain group except for .com extension (ICC = 0.43). The overall DISCERN score (based on average of all four raters) was highest for the website extension categories .edu (*m* = 20.13) and .gov (*m* = 16.19).

Lowest overall DISCERN score was assigned to the website extension category .com (*m* = 12.52) and can be attributed to limited information availability on COVID-19 treatment choices, treatment effect, objectives not defined clearly, poor information validity, and no or limited information on prevention strategies. The quality of information based on the overall DISCERN score was significantly different between the web domains (Table 2).

The DISCERN tool was broken down into six groups, and a score was assigned to each of the DISCERN category by the raters. An average DISCERN score for each DISCERN group for the websites is reported (Table 3). All raters consistently rated Objectives, Relevance, and Information Credibility of websites high. While quality of information on COVID-19 treatment choices and treatment effect consistently rated low by all the raters. The intracluster correlation coefficient value between 0 and 0.33 on all DISCERN tool categories demonstrates poor reliability across raters.

We calculated the overall DISCERN Objective score based on Objective score values of all the four raters. The procedure repeated for all the other DISCERN groups including Relevance, Information Credibility, Treatment Choices, Treatment Effect, and Prevention and Management and compared them across the different website domains (Table 4). As shown in the table, .com websites received the lowest average scores for website objectives, relevance, information credibility, treatment choices, treatment effect, and prevention and management. Websites in the .edu, category received the highest scores for objectives, relevance, information credibility, and prevention and management, indicating a better quality of novel coronavirus related health information. Among all .com website categories, objectives had the highest DISCERN score. Results were similar for objectives among .org and .gov website categories. Treatment choices scores were highest among the .edu website category. There were no significant differences in how the raters assigned DISCERN scores to the objectives and relevance categories (Table 4). However, significant difference among the raters for various other DISCERN score categories including information credibility, treatment choices, treatment effect, and prevention and management was seen across different website extensions. Results showed that .edu, .org, and other website categories were excellent in conveying novel coronavirus related health information; however, they received lower scores for treatment effect and treatment choices.

## 4. Discussion

The study results reveal that there is a lack of good quality websites with useful and quality novel coronavirus related health information. Results from sites like .edu, .gov, and .org and other website categories showed higher scores by the raters. The findings of the current study hold high importance in the current times as it identifies critical gaps in the information on novel coronavirus and other aspects of it. This information paves a way to understand that putting information out on the internet is not the basis for accurate information. The reliability of the information needs consideration, but prioritizing information personalization and tailoring according to the needs of the users is prime focus of information generation and further dissemination. The study's findings can guide users to choose between various websites and help the user prioritize the information available. Several studies have suggested that the internet can be a useful source of health information and assist patients and providers with clinical decision making [23–26]. The internet plays an important role in public health as it reaches a large part of the population and individuals increasingly turn to it for health information [8–27].

The ability to obtain online health information accurately, quickly, and conveniently offers consumers opportunity for better-informed decision making [8]. Searching useful and valid information on the internet can be difficult because of the speed and lack of control with which the information is accumulating. In this study, four independent raters who were frequent internet users were selected to search for information on the internet using the most common search engines. There were differences in how the quality of the information rated by the four independent raters. However, there could be various reasons for these differences, including prior knowledge and familiarity with the health information content. Another reason for this lower interrater reliability could be the subjective nature of some of the questions. It is important to consider the user's perspective when presenting health information content over the internet [9].

The internet is recognized as a basis to educate and empower patients. The internet can provide information on an individual's health problems, prevention/management of diseases, and related health services. The internet is perceived to have the ability to reach those with limited access to information on a wide breadth of topics, and access information when needed. However, prior studies have documented that the information on the internet is of poor quality. Future studies should employ more than one rater [28]. A prior study has recommended having three to four raters to evaluate the quality of health information on the internet and resolve any differences by consensus [29].

Voluntary organizations should regularly review information on their websites, specifically relating to the provision of up-to-date information on aspects of novel coronavirus and ensure quality of information on the website by providing author credentials and affiliations [30]. Variability in the quality of novel coronavirus related health information websites for core content points to the need for a grading system. This would allow individuals to reach trustworthy, up-to-date websites so that they can receive high-quality information to make informed decisions regarding prevention, treatment, and care. There are no clear universal guidelines governing healthcare information. Several approaches, such as trust marks that sites can display and principles that websites can use, should be used to regulate their behavior. Also, there should be a mechanism established that will evaluate if wrong info is being spread, that site should be closed down/some regulatory provisions should also be framed in this regard. One recommendation could be that websites, once ranked with trust marks, be popularized for better information dissemination. Opportunities should be created for public health experts and officials to work more effectively with local journalists to increase the impact of public health messages available on websites.

This study has several limitations. It provides only a snapshot in time of information represented in a rapidly changing medium. We expect that changes to the websites that evaluated would already alter some of the findings from the date of the search. Earlier studies have recommended that methods of indexing web pages improved and that information on the web needs to made more readable for users of different socioeconomic backgrounds [31].

The internet has the potential to be a powerful resource for meeting some of the public's health information needs. A shared responsibility between health information consumers and website developers would enable the design and development of targeted websites to address the needs of the individuals. Consumers are generally not aware of characteristics that indicate quality information on the internet [9]. Results of our study help in recognizing the websites that might be useful in gathering health information regarding novel coronavirus on the internet. Education of the users regarding the approach to find valid and authentic health information may make them informed about decision making regarding the information they are accessing.

## 5. Conclusion

This study has addressed the gaps in the quality of information that is available on the websites for the subject of novel coronavirus. Further, this study acknowledges and emphasizes the need for websites to develop and provide a clear statement of purpose. It also highlights the need for adequate pondering before this information becomes accessible to users to serve as a means of consumer empowerment instead of putting them in a state of confusion or misguidance. To rapidly disseminate and share newly acquired scientific evidence and experiences in understanding the disease and its control measures, the health informatics are important sources. It is important to analyze the information available on the internet regarding this disease. In any crisis, the informer and the consumer share equal responsibility to solve the problem and share only the right information. The novel coronavirus (COVID-19) pandemic is a case that depicts how accurate passage of health information at the accurate time can save lives while also improving the way we respond to outbreaks in general.

## Figures and Tables

**Table 1:** Categories and examples of the DISCERN tool.

DISCERN tool questions	Categories	Examples	No. of evaluation items
Are the aims clear?	Objectives	Introduction of coronavirus, diagnosis/assessment of coronavirus	2
Does it achieve its aims?			
Is it relevant?	Relevance	Risk factors/causes/ethology, symptoms of coronavirus	3
Is it clear what sources of information are used to compile the publication (other than the author or producer)?	Information credibility	Citations/reference material to the information presented	2
Is it clear when the information used or reported in the publication is produced?		Date of publication	
Is it balanced and unbiased?	Treatment choices	Product advertisement Coronavirus treatment and various treatment approaches How to self-manage coronavirus? Who to consult or refer to in case of coronavirus?	4
Is it clear that there may be more than one possible treatment choice?			
Based on the answers to all of the above questions, rate the overall quality of the publication as a source of information about treatment choices			
Does it describe how each treatment works?	Treatment effect	What are the best approaches evident for coronavirus treatment? What are the new treatment approaches that are currently being explored? Are the benefits of each treatment modality clearly illustrated? Complications of coronavirus	4
Does it describe the benefits of each treatment?			
Does it describe the risks of each treatment?			
Does it describe what would happen if no treatment is used?			
Does it describe how the treatment choices affect overall quality of life?			
Does it refer to areas of uncertainty?			
Does it provide support for shared decision-making?			
Does it provide details of additional sources of support and information?	Prevention and management	Who to consult or refer to?	1

**Table 2:** Total DISCERN scores (out of 30) by raters for each domain/extension category.

Extension category	*n*	Rater 1	Rater 2	Rater 3	Rater 4	Average score of all raters		ICC
		Mean (SD)				Mean (SD)	*p* value	
.com	22	14.41 (5.47)	11.59 (3.20)	11.82 (4.87)	12.27 (4.21)	12.52 (3.49)	0.0194	0.43
.edu	2	25 (0)	14.5 (0.71)	20.5 (0.71)	20.5 (4.95)	20.13 (20.13)		0.00
.gov	4	20.0 (3.74)	14.5 (5.0)	16.0 (11.05)	14.25 (7.14)	16.19 (14.13)		0.18
.org	9	22.56 (2.60)	12.12 (2.62)	13.89 (5.30)	14.23 (6.42)	15.69 (16.75)		0.12
Others	11	20.0 (5.93)	14.82 (4.64)	12.36 (5.48)	14 (6.54)	15.30 (15.75)		0.26

**Table 3:** Comparison of average DISCERN score (out of 5) of four raters on each group.

DISCERN group	Rater1	Rater2	Rater3	Rater4	ICC
	Mean (SD)	Mean (SD)	Mean (SD)	Mean (SD)	
Objectives	3.92 (1.30)	3.0 (0.88)	2.77 (1.32)	2.85 (1.30)	0.31
Relevance	3.85 (1.34)	2.75 (0.81)	2.60 (1.30)	2.60 (1.18)	0.28
Information credibility	3.56 (1.38)	2.52 (0.95)	2.50 (1.20)	2.52 (1.38)	0.33
Treatment choices	1.85 (0.92)	1.17 (0.38)	1.69 (0.99)	2.31 (1.22)	0.03
Treatment effect	1.27 (0.44)	1.15 (0.36)	1.08 (0.45)	1.46 (0.74)	0.00
Prevention	3.67 (1.49)	2.21 (0.94)	2.40 (1.28)	1.79 (1.24)	0.20

ICC, intracluster correlation coefficient.

**Table 4:** Average DISCERN score in each group by website category.

DISCERN group	.com	.edu	.gov	.org	Others	*p* value
	(*n* = 22)	(*n* = 2)	(*n* = 4)	(*n* = 9)	(*n* = 11)	
Objectives, mean (SD)	2.84 (0.87)	4.25 (0)	3.18 (0.72)	3.39 (0.86)	3.30 (0.97)	0.1612
Relevance, mean (SD)	2.61 (0.87)	3.88 (0.18)	3.13 (0.75)	3.25 (0.78)	3.16 (0.88)	0.1095
Information credibility, mean (SD)	2.34 (0.84)	3.88 (0.18)	3.13 (0.92)	3.11 (0.82)	3.05 (0.94)	0.0285
Treatment choices, mean (SD)	1.50 (0.39)	2.50 (0.35)	2.13 (0.83)	1.97 (0.40)	1.82 (0.53)	0.0074
Treatment effect, mean (SD)	1.15 (0.17)	1.75 (0.71)	1.56 (0.31)	1.31 (0.21)	1.16 (0.13)	0.0002
Prevention and management, mean (SD)	2.08 (0.67)	3.88 (0.18)	3.06 (0.97)	2.67 (0.77)	2.82 (1.05)	0.0079

## Data Availability

The data used to support the findings of this study are included within the article.

## Abbreviations

ANOVA: Analysis of variance
SD: Standard deviation
WHO: World Health Organization
CDC: Centers for Disease Control and Prevention
HON: Health on Net.

## Additional Points

What is already known? (i) The novel coronavirus (COVID-19) pandemic continues to spread across the United States and the world. There has been a lot of misinformation related to the novel coronavirus outbreak. The amount and nature of COVID-19 related information available to the public is changing and evolving constantly. Misinformation related to COVID-19 is recognized as a substantial public health challenge. (ii) Evidence-based and reliable health information of the disease from trustworthy sources is an urgent priority and a necessary step to disseminate accurate information to the public. (iii) Quality of health information related to COVID-19 requires ongoing evaluation. What are the new findings? (i) By using the quality of health information tools, we were able to demonstrate website extension categories rated as more reliable, with evidence-based information from trustworthy sources. (ii) It is also important to document information related to treatment choices as they become available for COVID-19 patients. What do the new findings imply? (i) The findings show that the quality of health information tools need to be applied to identify the most accurate and trusted sources of information related to COVID-19 outbreak. (ii) There is a critical need to develop a repository of reliable and evidence-based resources for the public at large. (iii) Future research and practice should focus on innovative population health informatics tools and technologies to disseminate COVID-19 health information in a format that is easy to understand.
